# Supplementary figures and images for: Root microbiota analysis of Oryza rufipogon and Oryza sativa reveals an orientation selection during the domestication process
Source: Microbiol Spectr. 2024 Mar 12;12(4):e03330-23. doi: 10.1128/spectrum.03330-23 (PMC10986595; doi:10.1128/spectrum.03330-23)

A

Relative Abundance

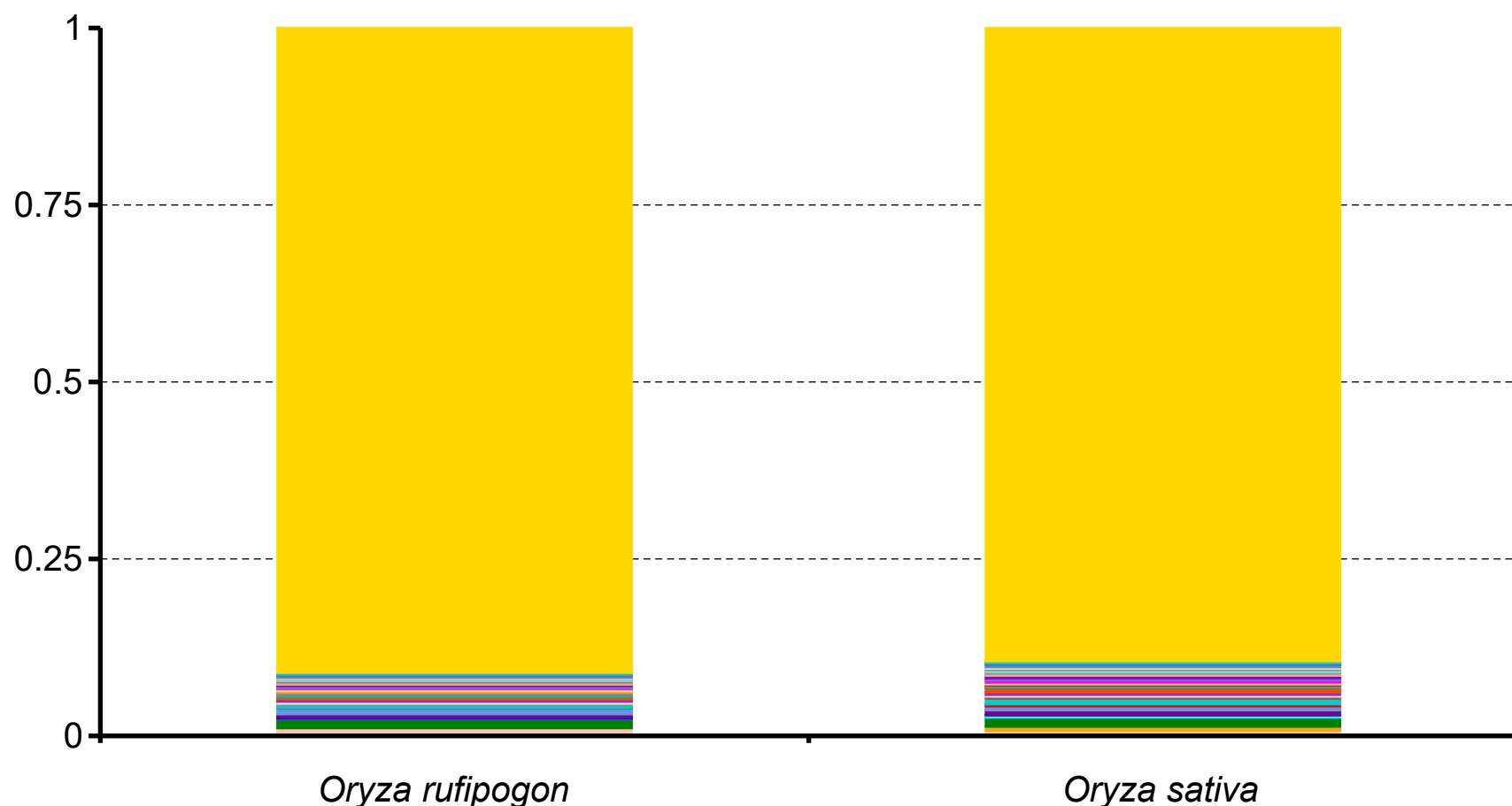

B

Relative Abundance

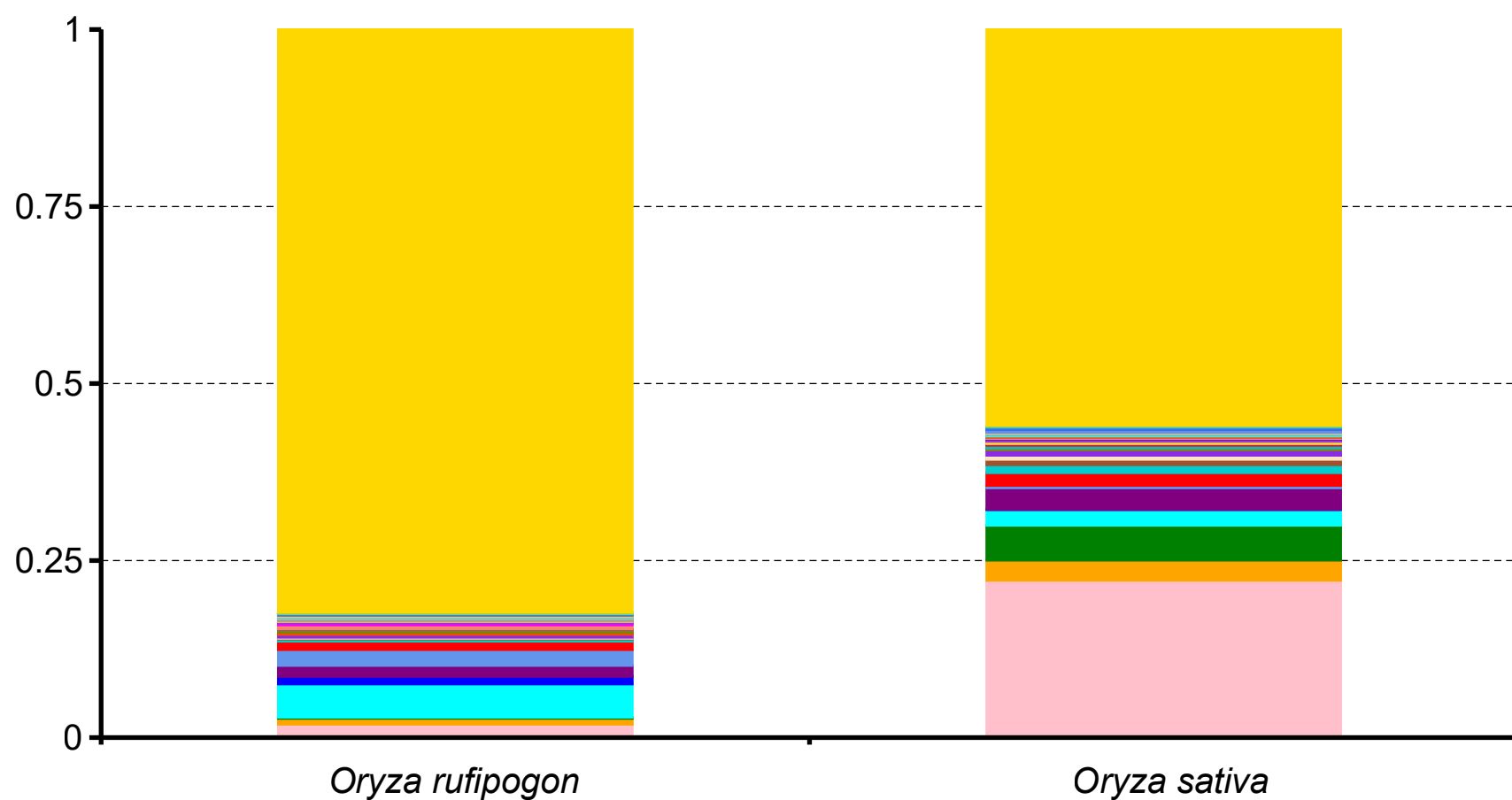

Supplement: Figure S1 — Relative abundance of bacterial and fungal composition at genus level of Oryza rufipogon and Oryza sativa. [file spectrum.03330-23-s0001.pdf]

A

*Oryza sativa**Oryza rufipogon*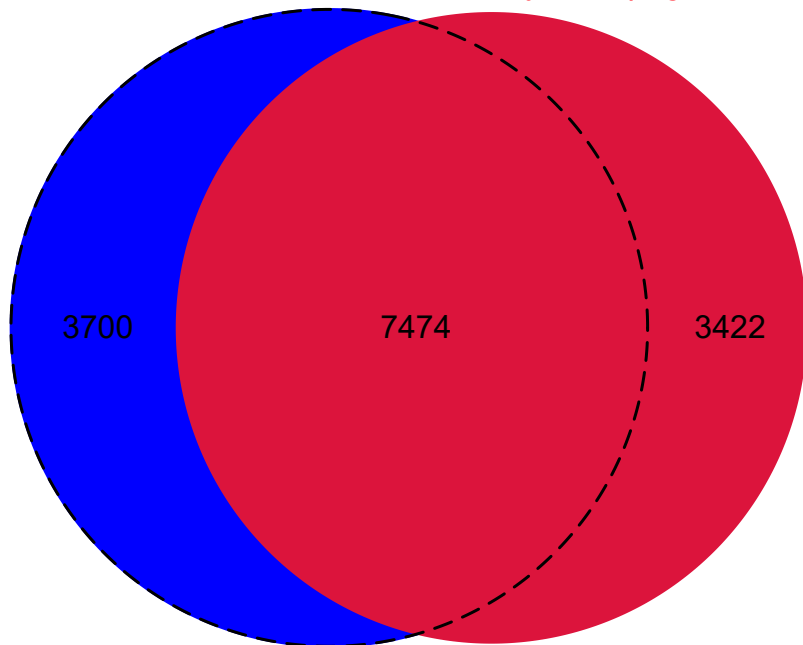

B

*Oryza rufipogon**Oryza sativa*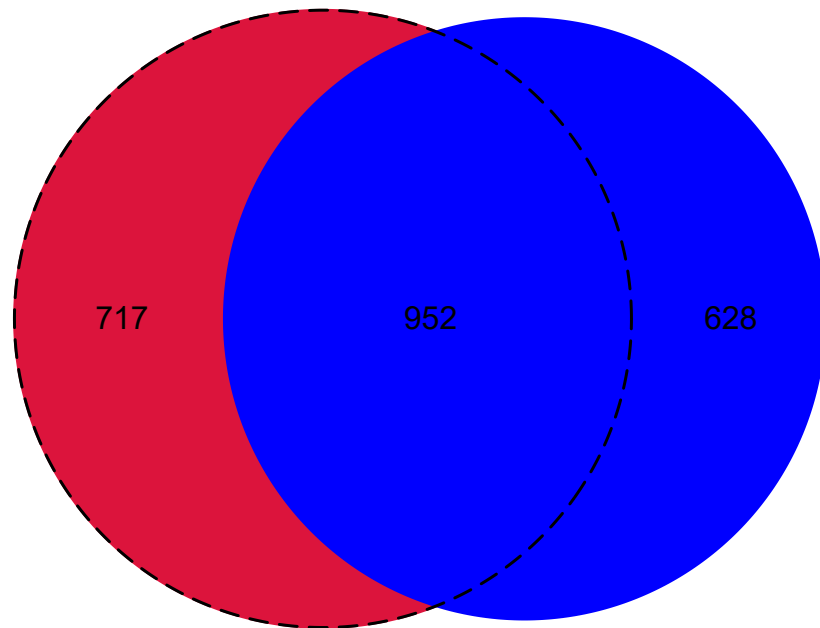

Supplement: Figure S2 — Venn diagram showing differences in bacterial and fungal community composition at OTU level of Orza rufipogon and Oryza sativa. [file spectrum.03330-23-s0002.pdf]

A

*Oryza sativa**Oryza rufipogon*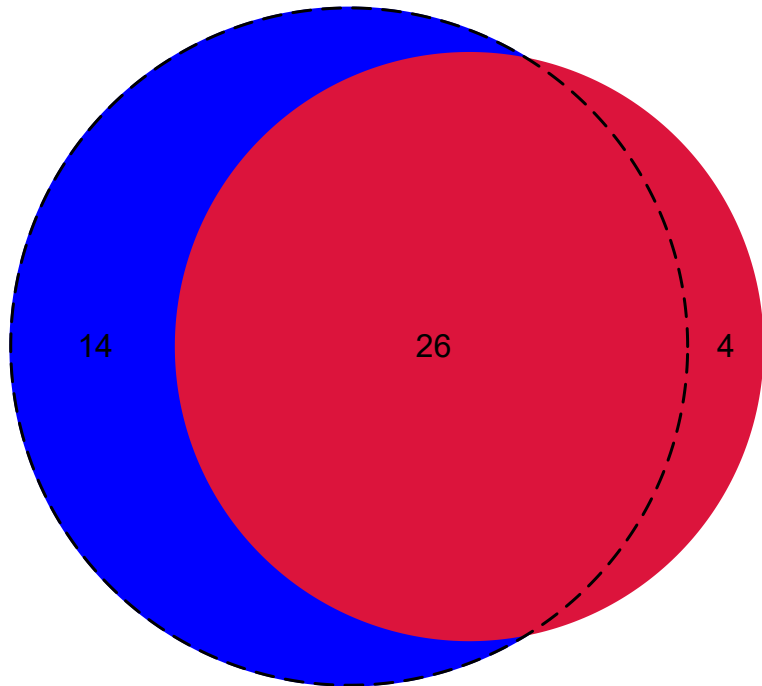

B

*Oryza sativa**Oryza rufipogon*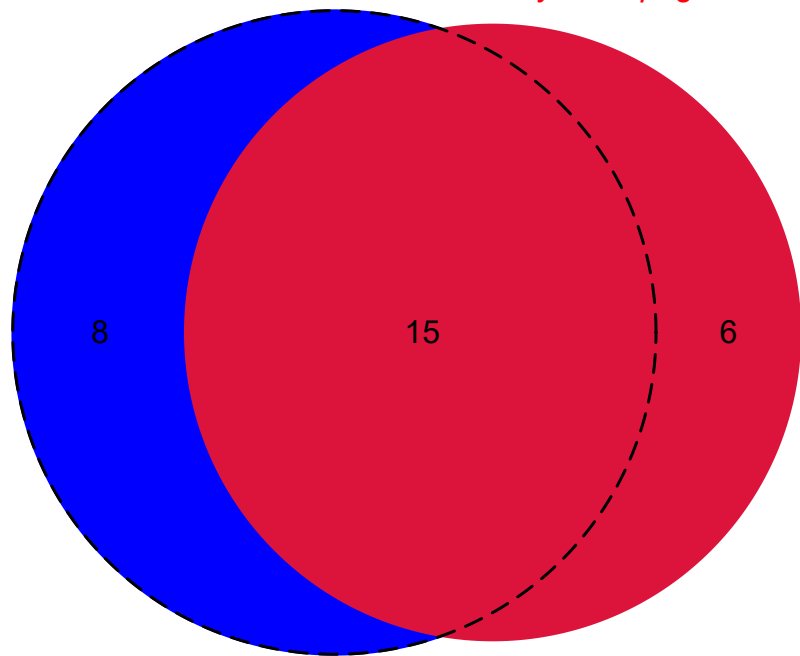

Supplement: Figure S3 — Venn diagram showing differences in core bacterial and fungal community composition at genus level of Oryza rufipogon and Oryza sativa. [file spectrum.03330-23-s0003.pdf]

A

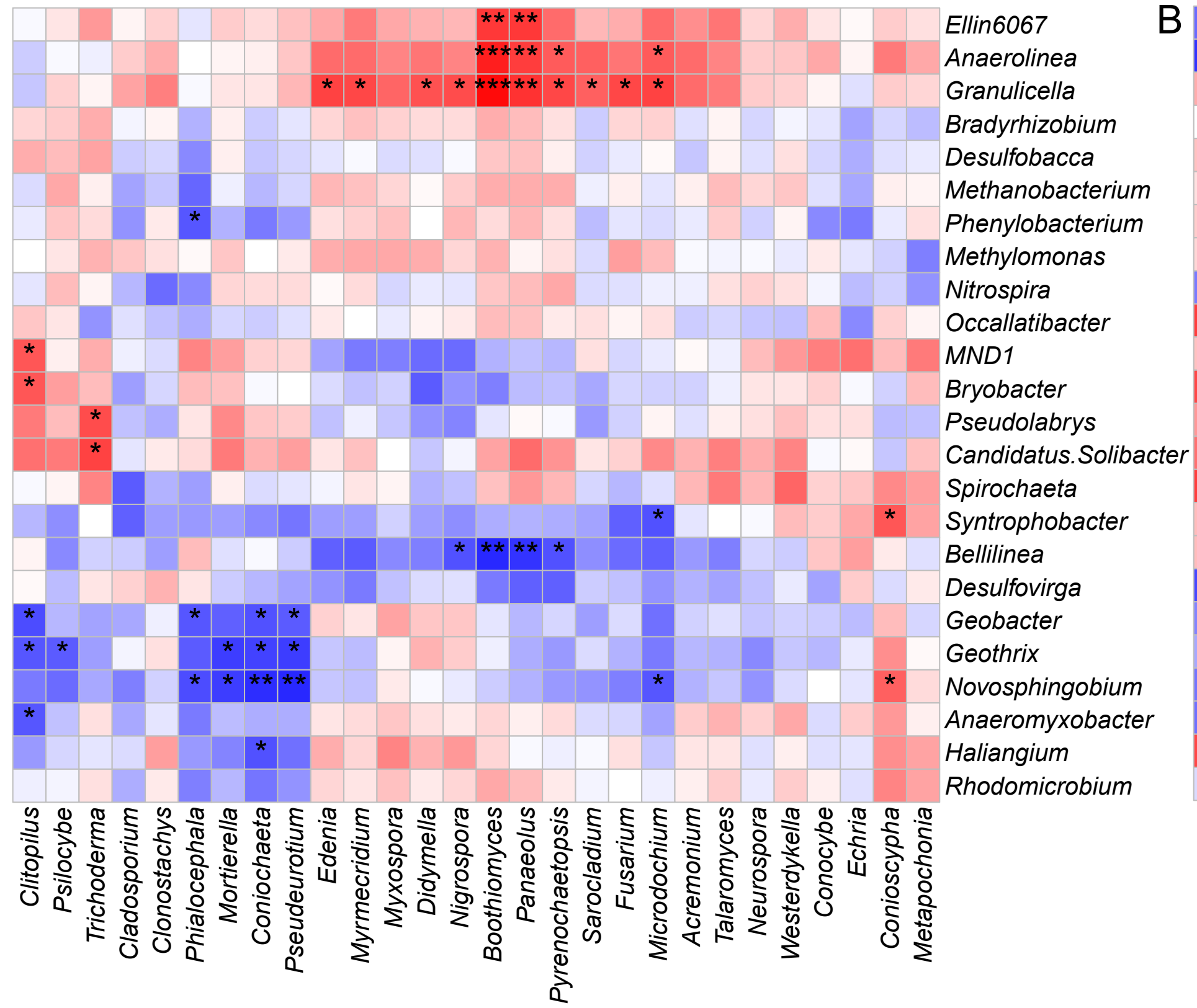

B

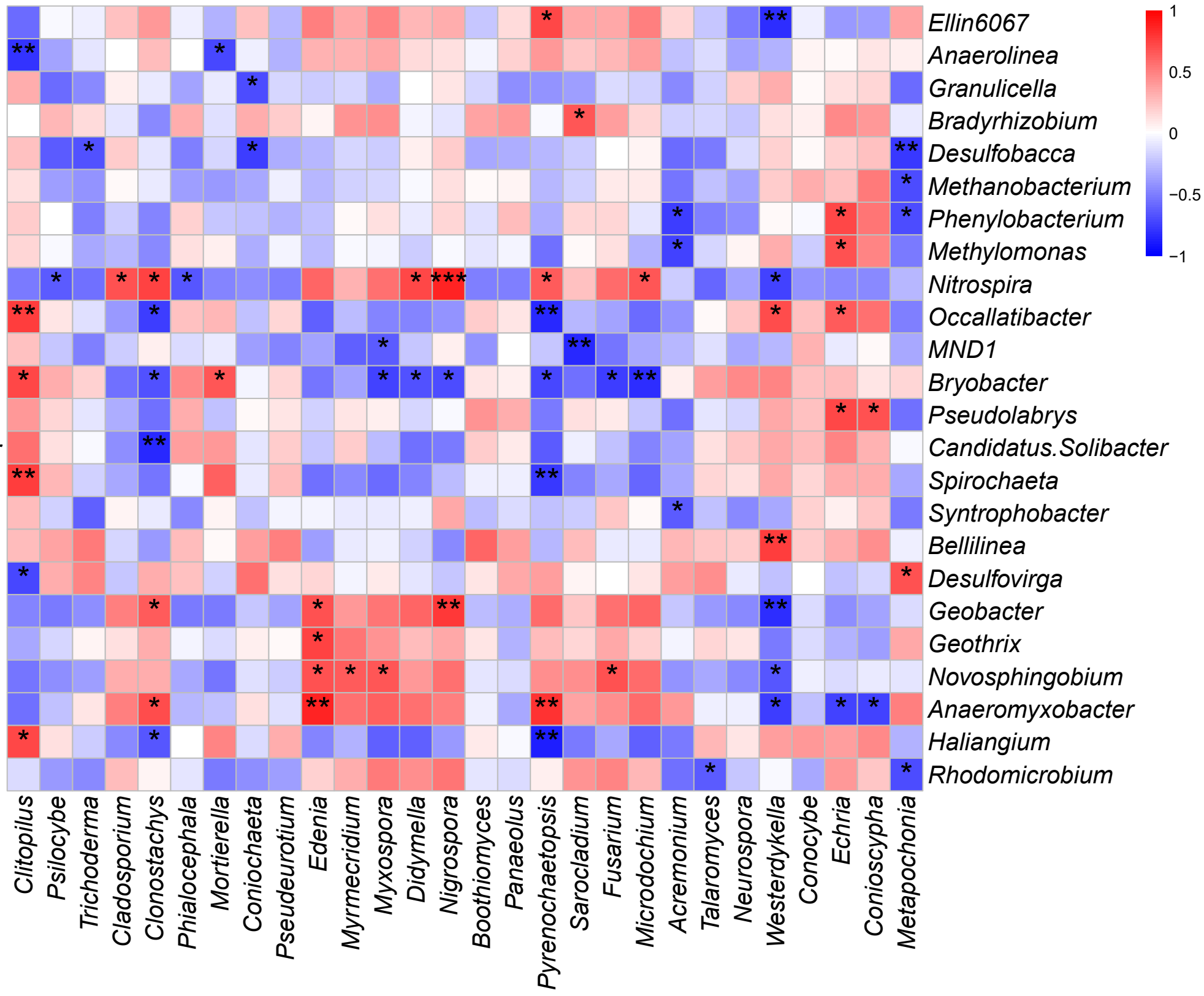

Supplement: Figure S4 — Spearman correlation of bacteria-fungi at genus level in Oryza rufipogon and Oryza sativa. [file spectrum.03330-23-s0004.pdf]
