# Supplementary material for: Root microbiota analysis of Oryza rufipogon and Oryza sativa reveals an orientation selection during the domestication process
Source: Microbiol Spectr. 2024 Mar 12;12(4):e03330-23. doi: 10.1128/spectrum.03330-23 (PMC10986595; doi:10.1128/spectrum.03330-23)
Supplement: Legends — to Fig. S1 to S4. [file spectrum.03330-23-s0005.docx]

**Supplementary Figure S1 (Fig. S1, see Supplementary Material).** Relative abundance of bacterial and fungal composition at genera level of *Oryza rufipogon* and *Oryza sativa*. **(A)** Bacteria. **(B)** Fungi.

**Supplementary Figure S2 (Fig. S2, see Supplementary Material).** Venn diagram showing differences in bacterial and fungal community composition at OTU level of *Orza rufipogon* and *Oryza sativa*. **(A)** Bacteria. **(B)** Fungi.

**Supplementary Figure S3 (Fig. S3, see Supplementary Material).** Venn diagram showing differences in core bacterial and fungal community composition at genera level of *Oryza rufipogon* and *Oryza sativa*. **(A)** Bacteria. **(B)** Fungi.

**Supplementary Figure S4 (Fig. S4, see Supplementary Material).** Spearman correlation of bacteria-fungi at genera level in *Oryza rufipogon* and *Oryza sativa*. Red represents positive correlation, blue represents negative correlation. The bigger and darker diamond represents higher correlation index. ‘*’ represents *p*-value<0.05, ‘**’ represents *p*-value<0.01, ‘***’ represents *p*-value<0.001. **(A)** *Oryza rufipogon*. **(B)** *Oryza sativa*.
