# Supplementary material for: Root microbiota analysis of Oryza rufipogon and Oryza sativa reveals an orientation selection during the domestication process
Source: Microbiol Spectr. 2024 Mar 12;12(4):e03330-23. doi: 10.1128/spectrum.03330-23 (PMC10986595; doi:10.1128/spectrum.03330-23)
Supplement: Table S1 — Sample information. [file spectrum.03330-23-s0006.pdf]

Table S1 Samples information

Specie	Sample Name	growth habit (1, up-right, angle from stem to ground ＞60°; 2, inclined, 30°＜angle from stem to ground＜60°; 3, crawl, angle from stem to ground ＜30°)	awn (0, non-awn; 1, short awns, length⩽2 cm; 2, partial short awns, length⩽2 cm; 3, partial long awns, length＞2 cm; 4, long awn, length＞2 cm)	leaf sheath color (1, yellow; 2, green; 3, purple)	height (the length from the ground to the top of the highest spike, centimeter, cm)	ligule color (0, non-ligule; 1, white; 2, light purple; 3, linear purple; 4, purple)
Oryza rufipogon	Or01	3	4	3	165.20	4
Oryza rufipogon	Or02	3	4	3	175.00	4
Oryza rufipogon	Or03	3	4	3	168.40	4
Oryza rufipogon	Or04	3	4	3	169.00	4
Oryza rufipogon	Or05	3	4	3	162.00	4
Oryza rufipogon	Or06	3	4	3	168.20	4
Oryza rufipogon	Or07	3	4	3	171.80	4
Oryza rufipogon	Or08	3	4	3	179.80	4
Oryza rufipogon	Or09	3	4	3	168.20	4
Oryza rufipogon	Or10	3	4	3	166.60	4
Oryza sativa	Os01	1	0	2	86.00	1
Oryza sativa	Os02	1	0	2	85.00	1
Oryza sativa	Os03	1	0	2	83.60	1
Oryza sativa	Os04	1	0	2	84.60	1
Oryza sativa	Os05	1	0	2	87.80	1
Oryza sativa	Os06	1	0	2	83.40	1
Oryza sativa	Os07	1	0	2	84.40	1
Oryza sativa	Os08	1	0	2	87.40	1
Oryza sativa	Os09	1	0	2	82.20	1
Oryza sativa	Os10	1	0	2	85.60	1
