# Supplementary material for: Root microbiota analysis of Oryza rufipogon and Oryza sativa reveals an orientation selection during the domestication process
Source: Microbiol Spectr. 2024 Mar 12;12(4):e03330-23. doi: 10.1128/spectrum.03330-23 (PMC10986595; doi:10.1128/spectrum.03330-23)
Supplement: Table S2 — Summary of the investigated samples in the current study. [file spectrum.03330-23-s0007.pdf]

Table S2 Summary of the investigated samples in the current study.

Sample_ID	Species	16S Effective Tags	ITS Effective Tags	16S Taxon Tags	ITS Taxon Tags	16S OTU_num	ITS OTU_num
Or01	*Oryza rufipogon*	98693	69043	33862	36531	4017	777
Or02	*Oryza rufipogon*	80455	48392	34090	37760	3658	701
Or03	*Oryza rufipogon*	86658	74497	33889	35740	3751	587
Or04	*Oryza rufipogon*	95043	89660	33742	39327	4186	447
Or05	*Oryza rufipogon*	82008	53491	33848	37814	3985	592
Or06	*Oryza rufipogon*	70237	50347	34052	39229	3902	554
Or07	*Oryza rufipogon*	78002	68602	33995	38368	3922	424
Or08	*Oryza rufipogon*	96255	96588	34019	36954	3536	296
Or09	*Oryza rufipogon*	65347	41624	32858	34913	3622	659
Or10	*Oryza rufipogon*	82753	68507	33746	39850	4209	617
Os01	*Oryza sativa*	85530	67123	33600	35846	4080	546
Os02	*Oryza sativa*	82384	68368	32934	35806	3801	542
Os03	*Oryza sativa*	73533	82720	33908	37117	4106	609
Os04	*Oryza sativa*	86017	61554	33408	39068	4086	579
Os05	*Oryza sativa*	63711	97745	33950	36267	3888	610
Os06	*Oryza sativa*	75897	45590	33921	34998	4198	474
Os07	*Oryza sativa*	67809	70694	33860	35130	4343	661
Os08	*Oryza sativa*	97943	97071	33795	38635	4375	439
Os09	*Oryza sativa*	94202	82374	33640	36290	4326	488
Os10	*Oryza sativa*	89043	83986	33806	35755	4187	476
